# Association of Maternal Lactation With Diabetes and Hypertension: A Systematic Review and Meta-analysis

**DOI:** 10.1001/jamanetworkopen.2019.13401

**Published:** 2019-10-16

**Authors:** Rabel Misbah Rameez, Divyajot Sadana, Simrat Kaur, Taha Ahmed, Jay Patel, Muhammad Shahzeb Khan, Sarah Misbah, Marian T. Simonson, Haris Riaz, Haitham M. Ahmed

**Affiliations:** 1 Department of Internal Medicine, Cleveland Clinic, Cleveland, Ohio; 2 Department of Pulmonary and Critical Care Medicine, Cleveland Clinic, Cleveland, Ohio; 3 Department of Internal Medicine, Cleveland Clinic–Fairview Hospital, Cleveland, Ohio; 4 Department of Internal Medicine, John H. Stroger Jr Hospital of Cook County, Chicago, Illinois; 5 Dow Medical College, Karachi, Pakistan; 6 Floyd D. Loop Alumni Library, Cleveland Clinic, Cleveland, Ohio; 7 Heart and Vascular Institute, Cleveland Clinic, Cleveland, Ohio; 8 AdvantageCare Physicians, Brooklyn, New York

## Abstract

**Importance:**

Lactation has been shown to be associated with lower rates of diabetes and hypertension in mothers. However, the strength of association has varied between studies, and sample sizes are relatively small.

**Objective:**

To conduct a systematic review and meta-analysis to determine whether lactation is associated with a lower risk of diabetes and hypertension.

**Data Sources:**

Ovid MEDLINE, Ovid Embase, Cochrane CENTRAL, and CINAHL databases were searched from inception to July 2018 with manual search of the references.

**Study Selection:**

Studies of adult women that specified duration of breastfeeding for at least 12 months, evaluated primary hypertension and diabetes as outcomes, were full-text articles in English, and reported statistical outcomes as odds ratios were included.

**Data Extraction and Synthesis:**

Study characteristics were independently extracted using a standard spreadsheet template and the data were pooled using the random-effects model. The Meta-analysis of Observational Studies in Epidemiology (MOOSE) guideline for reporting was followed.

**Main Outcomes and Measures:**

Diabetes and hypertension.

**Results:**

The search yielded 1558 articles, from which a total of 6 studies met inclusion criteria for association between breastfeeding and diabetes and/or hypertension. The 4 studies included in the meta-analysis for the association between lactation and diabetes had a total of 206 204 participants, and the 5 studies included in the meta-analysis for the association between lactation and hypertension had a total of 255 271 participants. Breastfeeding for more than 12 months was associated with a relative risk reduction of 30% for diabetes (pooled odds ratio, 0.70 [95% CI, 0.62-0.78]; *P* < .001) and a relative risk reduction of 13% for hypertension (pooled odds ratio, 0.87 [95% CI, 0.78-0.97]; *P* = .01).

**Conclusions and Relevance:**

This study suggests that education about the benefits of breastfeeding for prevention of diabetes and hypertension in women is a low-risk intervention that can be easily included in daily practice and may have a positive impact on cardiovascular outcomes in mothers.

## Key Points

**Question:**

Is breastfeeding associated with lower risk of maternal diabetes or hypertension?

**Findings:**

This meta-analysis of 6 studies including more than 200 000 participants found that breastfeeding was associated with a relative risk reduction of 30% for diabetes and 13% for hypertension in the mothers studied.

**Meaning:**

These findings suggest that breastfeeding is associated with long-term cardiovascular health benefits for women.

## Teaser

This systematic review and meta-analysis examines the association between breastfeeding and maternal risk of diabetes and hypertension.

## Introduction

Atherosclerotic cardiovascular diseases are the leading cause of death globally, with an estimated 17.3 million deaths per year.^1^ Hypertension and diabetes are associated with an increased risk of atherosclerotic cardiovascular diseases and cardiovascular mortality,^2^ and presence of diabetes may be considered a coronary artery disease equivalent in terms of cardiovascular risk.^3^ Moreover, both diabetes and hypertension independently account for the 7th and 13th leading causes of death in the United States, respectively.^4^

Cardiovascular disease remains the leading cause of death in women as well.^5^ Women share many of the traditional risk factors for cardiovascular disease; however, they also have unique cardiovascular and metabolic stresses in the setting of pregnancy and the puerperium.^5^ Lactation has been thought to be associated with positive effects on the postpartum state and is thought to work as a physiological reset to the adverse effects of pregnancy.^6^ However, sample sizes from various studies of lactation and cardiovascular risk have been relatively small, and there is disparity in the outcomes reported. The aim of this systematic review and meta-analysis was to determine whether lactation is associated with reduced rates of maternal diabetes and hypertension.

## Methods

This systematic review and meta-analysis followed the Meta-analysis of Observational Studies in Epidemiology (MOOSE) reporting guideline and the American Heart Association guideline.^7,8^ As a meta-analysis, the study was considered exempt by our institutional review board.

### Data Sources and Search Strategy

The search strategy for electronic databases was developed in conjunction with a medical librarian experienced in systematic reviews. Electronic searches were performed in July 2018 in the following databases: Ovid MEDLINE, Ovid Embase, Cochrane CENTRAL, and CINAHL Plus.

A combination of Medical Subject Heading terms, other controlled vocabulary, and keywords were used to search reports published in English for “cardiovascular diseases,” “metabolic syndrome,” “type 2 diabetes,” “risk factors,” “breastfeeding,” and “women.” In addition, we manually searched reference lists of selected articles to find any other relevant citations that were not detected by the electronic searches. The complete search strategies for each database are provided in the eAppendix in the Supplement.

Two of us (R.M.R. and S.K.) independently extracted data and were blinded. Disputes were resolved by mutual discussion or by a third investigator (H.M.A.). Relevant articles were initially selected on the basis of the title and abstract, after which the full text was read to confirm relevance. The reference lists of the retrieved articles and the relevant reviews were then screened to identify pertinent studies.

### Study Selection

We used the following inclusion criteria: studies of adult women that specified duration of breastfeeding for at least 12 months, investigated primary hypertension and/or diabetes as outcomes, were full-text articles in the English language, and reported statistical outcomes as odds ratios (ORs) adjusted for confounding variables. However, we collected data from studies that reported results as unadjusted ORs, relative risk (RR), or hazard ratios (HRs) to use in a separate subanalysis. Exclusion criteria included studies that did not specify duration of breastfeeding, those that studied breastfeeding duration for less than 12 months, or those that reported gestational diabetes or other gestational disorders only, such as preeclampsia or eclampsia. Although we made an effort to qualitatively review studies that reported outcomes as raw data, we did not include them in the final meta-analysis because it was not possible to adjust the calculated OR RR for confounding variables in each of these studies.

### Data Extraction and Quality Assessment

The following data were extracted by 2 of us (R.M.R. and S.K.) on a standardized data collection form: name of authors, study name, region, year of publication, journal, type of study, total number of participants, age group of participants, follow-up, duration of breastfeeding, primary outcome (hypertension or diabetes), statistical outcome used (OR, RR, or HR; both adjusted and unadjusted when available), and variables adjusted for in each study. Where data were missing or clarifications were needed, the authors were contacted. Quality of the included studies was appraised using the standardized Newcastle-Ottawa Scale (eTable 1 in the Supplement).^9^

### Statistical Analysis

The principal summary statistic was OR with 95% confidence intervals. To account for potential study variance, we performed a DerSimonian-Laird random-effects model meta-analysis of extracted data using the metan package in Stata statistical software version 15.1 (StataCorp).^10^ Cochrane *Q* statistics and *I*^2^ tests were used to assess for heterogeneity. Values of 25%, 50%, and 75% were considered low, moderate, and high degrees of heterogeneity, respectively.^11^ Statistical significance was assumed at *P* < .05 using 2-tailed tests. To assess for publication bias, an Egger test was used.^12,13^ We did not use a funnel plot for this purpose because fewer than 10 studies were included.

## Results

### Literature Search

The initial literature search yielded a total of 1558 records; after removing 442 duplicates, 1116 studies remained (eFigure 1 in the Supplement). The reviewers screened the titles and abstracts of the 1116 studies, and a total of 107 full-text articles were reviewed for eligibility. Twenty-two studies went through a qualitative analysis, and 6 of the studies met full inclusion criteria and underwent quantitative synthesis or meta-analysis.^14,15,16,17,18,19^ A Preferred Reporting Items for Systematic Review and Meta-Analyses statement (PRISMA) flowchart^20^ is provided in eFigure 1 in the Supplement.

### Systematic Review of 22 Studies

The details and characteristics of the 22 studies^14,15,16,17,18,19,21,22,23,24,25,26,27,28,29,30,31,32,33,34,35,36,37^ included in the qualitative analysis are shown in eTable 2 in the Supplement. Each study was designed differently. The 16 studies that were not included in the final meta-analysis still provided valuable information in support of the results, so we discuss them here.

Bajaj et al^32^ found that the prevalence of diabetes was lower in women who had breastfed for more than 12 months compared with women who had breastfed for less than 3 months. Liu and colleagues^14^ used data from the Australian 45 and Up Cohort Study. The authors found that total breastfeeding duration and duration per child was associated with a reduction in the risk of development of diabetes by approximately 14% per year of breastfeeding. Schwarz et al^15^ concluded that breastfeeding for 12 or more months was associated with a decreased risk of hypertension, diabetes, hyperlipidemia, and cardiovascular disease. Unusually, the study population included women aged 50 to 79 years who were followed up for 7.9 years. On closer inspection, however, it was found that in addition to prospectively analyzing outcomes, retrospective analysis of data gathered on recruitment was also performed. In a cross-sectional study, Zhang and colleagues^16^ showed that women who breastfed were less likely to develop hypertension and diabetes. This remained significant when results were analyzed based on breastfeeding intervals of 0 to 6, 6 to 12, and more than 12 months, as well as when results were adjusted for confounding variables.

Choi and colleagues^17^ showed that breastfeeding for 12 or more months was associated with a lower risk of diabetes and metabolic syndrome. The Shanghai Women’s Health Study^24^ found that not only did women who breastfed have a lower risk of diabetes, increasing duration of breastfeeding was associated with a lower risk as well. Similarly, Jäger et al^30^ concluded that breastfeeding for 6 or more months may be associated with a lower risk of diabetes. Breastfeeding was associated with protective effects against the development of atherosclerotic disease, with shorter duration of lactation associated with subclinical atherosclerosis as determined by measuring carotid intima media thickness.^33^ Stuebe et al^23^ found that at 3 years post partum, women who breastfed for more than 6 months had lower weight retention. However, in a multivariate analysis, no consistent trend was found relating the association between breastfeeding and maternal metabolism in general. Schwarz and colleagues^21^ compared parous women who breastfed for 1 month or more and nulliparous women, and concluded that parous women who never breastfed had a higher risk of developing diabetes. Interestingly, women who engaged in exclusive breastfeeding for 1 to 3 months had a lower risk of developing diabetes compared with those who engaged in nonexclusive breastfeeding.

Breastfeeding initiation was associated with a reduced risk of diabetes in women with and without a history of gestational diabetes in a study by Martens et al.^35^ This association also remained significant after adjusting for confounding variables. Kirkegaard and colleagues^26^ concluded there was a strong, graded inverse association between lactation and incidence of diabetes when looking at various breastfeeding durations ranging from 0 to 6 months, 6 to 12 months, and longer than 12 months. Similarly, the Study of Women’s Health Across the Nation (SWAN)^36^ found that breastfeeding was associated with a lower prevalence of diabetes in a dose-response manner as well.

Using data from the Nurses’ Health Study, a large prospective cohort with more than 44 000 participants, Stuebe et al^29^ found that exclusive breastfeeding for longer than 6 months or total breastfeeding for longer than 12 months was associated with a lower risk of developing hypertension later in life compared with no breastfeeding or breastfeeding for less than 6 months. These findings remained significant after adjusting for confounders. Similarly, the Korean Women’s Study^27^ suggested that breastfeeding for 1 to 6 months or longer was associated with a lower risk of hypertension compared with no history of lactation. This study also found that the combination of obesity and gestational hypertension was associated with a higher risk of developing hypertension.

Park and Choi^28^ found that a greater number of breastfed children and longer duration of breastfeeding were associated with a lower risk of hypertension. This association was moderated by the degree of obesity and insulin resistance. A study by Kirkegaard et al^26^ studied the association between breastfeeding and hypertension as well as the development of cardiovascular disease and found that longer duration of breastfeeding was associated with a lower risk of hypertension and cardiovascular disease. Kim and Kim^25^ showed that any breastfeeding was better than none when assessing the association between breastfeeding and metabolic syndrome. Interestingly, a cross-sectional study^31^ of more 900 women in Iran did not support the protective associations of breastfeeding with development of metabolic syndrome.

Chetwynd et al^18^ used data from the Black Women’s Health Study and found that breastfeeding was associated with reduced risk of hypertension at ages 40 to 49 years, but not necessarily at older ages. Increasing the duration of breastfeeding was associated with a lower risk of developing hypertension in this age group, with the strongest association seen in women who breastfed for a cumulative time of 24 months or more. Lupton and colleagues^19^ used data from the Australian 45 and Up Study. Data from more than 74 000 women aged 45 years and older were analyzed to determine the association of parity and breastfeeding with maternal hypertension. The authors observed that lifetime breastfeeding for longer than 6 months or more than 3 months per child was associated with lower odds of having high blood pressure.

### Meta-analysis of 6 Final Studies

Six studies^14,15,16,17,18,19^ reported diabetes or primary hypertension as an outcome and were included in the meta-analysis (4 reported diabetes as the outcome and 5 reported primary hypertension as the outcome). The 4 studies^14,15,16,17^ included in the final meta-analysis for the association between lactation and diabetes had a total of 206 204 participants. The 5 studies^15,16,17,18,19^ that were included in the meta-analysis for association between lactation and hypertension included a total of 255 271 women. Characteristics of the included studies are shown in the Table. For breastfeeding and risk of either hypertension or diabetes after 12 months, an Egger test revealed *P* values of .51 and .93, respectively, indicating that publication bias did not alter the results. Mean (range) follow-up duration was 9.6 (3-18) years.

**Table.** Characteristics of Studies Included in the Meta-analysis for Hypertension and Diabetes

Source	Type of Study	Total No. of Participants	Follow-up, y	Age Group, y	Region or Country	Race/Ethnicity	Outcome	Outcome Assessment	Adjusted Covariates	Inclusion Criteria	Exclusion Criteria
Zhang et al,^16^ 2015	Cross-sectional	9128	NA	40-81	China	Asian	Hypertension and diabetes	Measured or, if self-reported, confirmed by review of medical records	Age, BMI, waist-hip ratio, working status, educational level, smoking, alcohol use, family history, age at menarche and/or menopause, age at childbearing, postpartum BMI	1 Lifetime birth	NA
Choi et al,^17^ 2017	Cross-sectional study using a multistage, stratified sampling method	4724	3	19-50	Korea	Asian	Hypertension and diabetes	Measured or, if self-reported, confirmed by review of medical records, blood tests, or use of medication	Age, BMI, household income, educational level, marriage status, smoking status, alcohol drinking, physical activity, age at menarche, menopause, parity, and use of oral contraceptives	Parous women aged 19-50 y	Current pregnancy, no history of pregnancy, incomplete analytic data
Lupton et al,^19^ 2013	Cross-sectional	64 199	NA	45-64	Australia	Australian, 0.9% Aboriginal or Torres Strait Islander origin	Hypertension	Self-reported, being treated within the last month	Age, country of origin, income, family history of hypertension, BMI, smoking status, alcohol use, physical activity, oral contraceptive use, hormone replacement therapy use, No. of children	Nulliparous or parous between ages 18-45 y, current age ≥45 y	History of gestational hypertension
Chetwynd et al,^18^ 2017	Nested case-control	37 539	18	40-49	United States	African American	Hypertension	Physician diagnosed or currently using medication	Age, survey cycle, parity, age at first birth, diet, exercise, BMI at age 18 y, smoking, family history of myocardial infarction	Parous women, response to questions about lactation history	Onset of hypertension before age 40 y, age ≥40 y at birth of last child, age >65 y at time of diagnosis
Schwarz et al,^15^ 2009	Observational, prospective cohort with retrospective analysis of data	139 681	7.9	50-79	United States	White, Hispanic, Latin American, Pacific Islander, Asian, Native American; inclusion of 20% of women from minority groups	Hypertension and diabetes	Measured or, if self-reported, confirmed by a physician, review of medical records	Age, race, parity, age at menopause, education, income, family history, physical activity, energy, diet intake, tobacco use history, hormone replacement therapy, aspirin use, multivitamin use, BMI	Postmenopausal, ≥1 live birth	Competing risks, eg, cancer; safety reasons (eg, severe disease); nulliparous; stillbirth history; missing data
Liu et al,^14^ 2010	Cross-sectional	52 731	NA	>45	Australia	NA	Diabetes	Self-reported	Age, BMI, smoking, alcohol consumption, physical activity, family history of diabetes, household income, education level, country of birth, and No. of births	Women recruited in the 45 and Up Study who answered questionnaires on sociodemographic and reproductive factors	Diabetes diagnosed before age 31 y or before the age when women last gave birth, or with unknown age at diagnosis; unknown parity and unknown breastfeeding status

Abbreviations: BMI, body mass index (calculated as weight in kilograms divided by height in meters squared); NA, not available.

### Diabetes Risk After 12 Months of Breastfeeding

**Figure 1.**
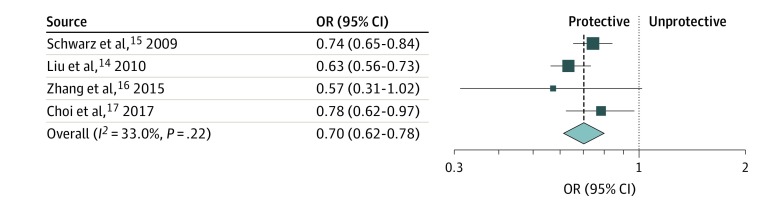
Meta-analysis Showing Association Between Breastfeeding and Diabetes Breastfeeding for 12 months or more was associated with a reduced risk of diabetes. The size of the data markers indicates the weight of the odds ratio (OR), using random-effects analysis with instrumental variables.

For meta-analysis of the outcomes, we used adjusted ORs to account for confounding by variables such as obesity, smoking, and family history on the association of breastfeeding with hypertension and diabetes. We observed that breastfeeding for longer than 12 months was associated with a relative risk reduction of 30% for diabetes compared with breastfeeding for less than 12 months, with a pooled OR of 0.70 (95% CI, 0.62-0.78; *P* < .001; *I*^2^ = 33%) (Figure 1). We found no difference in the results when we pooled together the results of studies reporting RR, OR, and HR (eFigure 2 and eFigure 3 in the Supplement).

### Hypertension Risk After 12 Months of Breastfeeding

**Figure 2.**
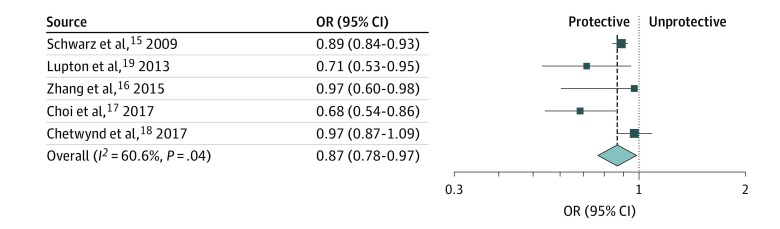
Meta-analysis Showing Association Between Breastfeeding and Hypertension Breastfeeding for 12 months or more was associated with a reduced risk of hypertension. The size of the data markers indicates the weight of the odds ratio (OR), using random-effects analysis with instrumental variables.

Breastfeeding for longer than 12 months was associated with a relative risk reduction of 13% for hypertension compared with breastfeeding for less than 12 months (pooled OR, 0.87 [95% CI, 0.78-0.97]; *P* = .01; *I*^2^ = 60.6%) (Figure 2). Again, no difference in results was found when we conducted a pooled analysis of studies that reported RR and OR (eFigure 4 in the Supplement).

## Discussion

The results of this meta-analysis with more than 200 000 participants suggest that breastfeeding for longer than 12 months is associated with decreased maternal risk of developing hypertension and diabetes. This was true even after adjusting for traditional cardiovascular confounders, as we used the adjusted summary estimates from each study. Cardiovascular disease is the leading cause of death in women,^38^ and hypertension and diabetes are both preventable diseases that are strong risk factors associated with atherosclerotic cardiovascular diseases.^39^ Lifestyle modifications such as weight loss, smoking cessation, reduction in alcohol intake, exercise, and diet are well-established means of reducing risk of cardiovascular disease and stroke.^40^

Pregnancy is associated with an adverse metabolic profile. Pregnant women are more likely to have an atherogenic lipid profile owing to an elevation of total cholesterol, low-density lipoprotein cholesterol, and triglyceride levels, which are essential for the nutrition of the developing fetus. Pregnancy is also associated with insulin resistance and glucose intolerance. Insulin resistance and glucose intolerance increase as part of a normal pregnancy. A 2007 review found that nonobese pregnant women have a 44% increase in insulin resistance by 36 weeks of gestation.^41^ By slowing maternal glucose absorption and increasing fat reserves, the fetus is preferentially supported.^42^

Lactation results in consumption of 500 calories per day.^17^ Breast milk is rich in cholesterol and mobilizes fat stores, enhances catabolism, and increases high-density lipoprotein levels. It has been thought that breastfeeding is a reset mechanism to the adverse metabolic profile in pregnancy, so women who do not breastfeed may be at risk for a persistently dysmetabolic state.^42^ Several studies have shown that lactation is associated with a decreased risk of diabetes and hypertension after adjusting for confounding variables such as age, smoking, obesity, and family history.^42^

Oxytocin is a neuropeptide that plays an important role in breastfeeding and uterine contractions during childbirth. It has also been associated with reduced stress, vascular resistance, and decreased blood pressure and may play a critical role in the association between lactation and decreased blood pressure post partum in women who breastfeed.^43,44^

In addition to diabetes and hypertension, breastfeeding has been associated with a lower risk of cardiovascular disease,^45,46,47^ obesity,^48^ and metabolic syndrome^17,36^ as well as decreased overall cardiovascular mortality^49^ in women in several studies, which demonstrates the great potential for further research in this area.

### Limitations

Our study faced limitations inherent to meta-analysis studies, which use pooled data without access to original patient data. None of the studies that we used were randomized clinical trials, so there may be an element of confounding bias. We used adjusted ORs because there are many confounding factors associated with cardiovascular outcomes, such as obesity, socioeconomic factors, smoking, and family history, in each of the included studies. However, there was heterogeneity for the confounding variables that each study adjusted for. Another issue is that the ascertainment of lactation history was usually self-reported in the studies using structured interviews, questionnaires, or open-ended questions, which could have led to recall bias as many studies were conducted years after the participants had given birth. None of the studies included reported blinding in any way to the exposure of interest (lactation). Outcome assessment was not always validated by record linkage or objective assessment, with some studies using an individual’s report of having hypertension or diabetes.

There was variability in the follow-up times in each study, which ranged from 3 to 18 years, and none of the studies reported the point for the development of outcome of interest. This does raise uncertainty about the proportion of association that breastfeeding has with the development of diabetes and hypertension over time. Additionally, owing to the heterogeneity of the statistical outcomes reported in studies, which included HR, OR, and RR, we had to exclude a number of studies from the final meta-analysis. On balance, all the studies analyzed showed a signal toward benefit of lactation, and the pooled outcomes and subanalysis that included studies that otherwise met inclusion criteria but reported results as RR and HR showed a strong, statistically significant protective association with little heterogeneity.

## Conclusions

Breastfeeding for longer than 12 months was associated with a 30% lower risk of diabetes and a 13% lower risk of hypertension in mothers after adjusting for confounding variables. The prenatal and antenatal period is an important opportunity to educate women about lifestyle interventions that may protect their health in the future. In addition to weight loss, smoking cessation, and exercise, breastfeeding should also be recommended owing to its benefits for the mother. Because this meta-analysis showed association but not causation, further research is needed to better understand this result. However, given the low-risk nature of this intervention, educating mothers about the potential benefits of breastfeeding for their cardiovascular health can be easily introduced into clinical practice when addressing prevention of cardiovascular outcomes in women.

## Notes

Supplement. **eFigure 1.** PRISMA Flow Chart Illustrating Results of the Literature **eTable 1.** Assessment of Quality of Studies Using the Newcastle-Ottawa Scale **eFigure 2.** Subanalysis With Studies Reporting RR, OR and Unadjusted OR for Breastfeeding and Diabetes **eFigure 3.** Subanalysis With Studies Reporting RR, OR, Unadjusted OR and HR for Breastfeeding and Diabetes **eFigure 4.** Subanalysis With Studies Reporting RR, OR and Unadjusted OR for Breastfeeding and Hypertension **eAppendix.** Detailed Search Strategy **eTable 2.** Qualitative Analysis of 18 Studies
