# Supplementary material for: Association of Maternal Lactation With Diabetes and Hypertension: A Systematic Review and Meta-analysis
Source: JAMA Netw Open. 2019 Oct 16;2(10):e1913401. doi: 10.1001/jamanetworkopen.2019.13401 (PMC6806428; doi:10.1001/jamanetworkopen.2019.13401)
Supplement: Supplement. — eFigure 1. PRISMA Flow Chart Illustrating Results of the Literature eTable 1. Assessment of Quality of Studies Using the Newcastle-Ottawa Scale eFigure 2. Subanalysis With Studies Reporting RR, OR and Unadjusted OR for Breastfeeding and Diabetes eFigure 3. Subanalysis With Studies Reporting RR, OR, Unadjusted OR and HR for Breastfeeding and Diabetes eFigure 4. Subanalysis With Studies Reporting RR, OR and Unadjusted OR for Breastfeeding and Hypertension eAppendix. Detailed Search Strategy eTable 2. Qualitative Analysis of 18 Studies [file jamanetwopen-2-e1913401-s001.pdf]

Supplementary Online Content

Rameez RM, Sadana D, Kaur S, et al. Association of maternal lactation with diabetes and hypertension: a systematic review and meta-analysis. *JAMA Netw Open*. 2019;2(10):e1913401. doi:10.1001/jamanetworkopen.2019.13401

eFigure 1. PRISMA Flow Chart Illustrating Results of the Literature

eTable 1. Assessment of Quality of Studies Using the Newcastle-Ottawa Scale

eFigure 2. Subanalysis With Studies Reporting RR, OR and Unadjusted OR for Breastfeeding and Diabetes

eFigure 3. Subanalysis With Studies Reporting RR, OR, Unadjusted OR and HR for Breastfeeding and Diabetes

eFigure 4. Subanalysis With Studies Reporting RR, OR and Unadjusted OR for Breastfeeding and Hypertension

eAppendix. Detailed Search Strategy

eTable 2. Qualitative Analysis of 18 Studies

This supplementary material has been provided by the authors to give readers additional information about their work.

© 2019 Rameez RM et al. *JAMA Network Open.*

eFigure 1: PRISMA flow chart illustrating results of the literature

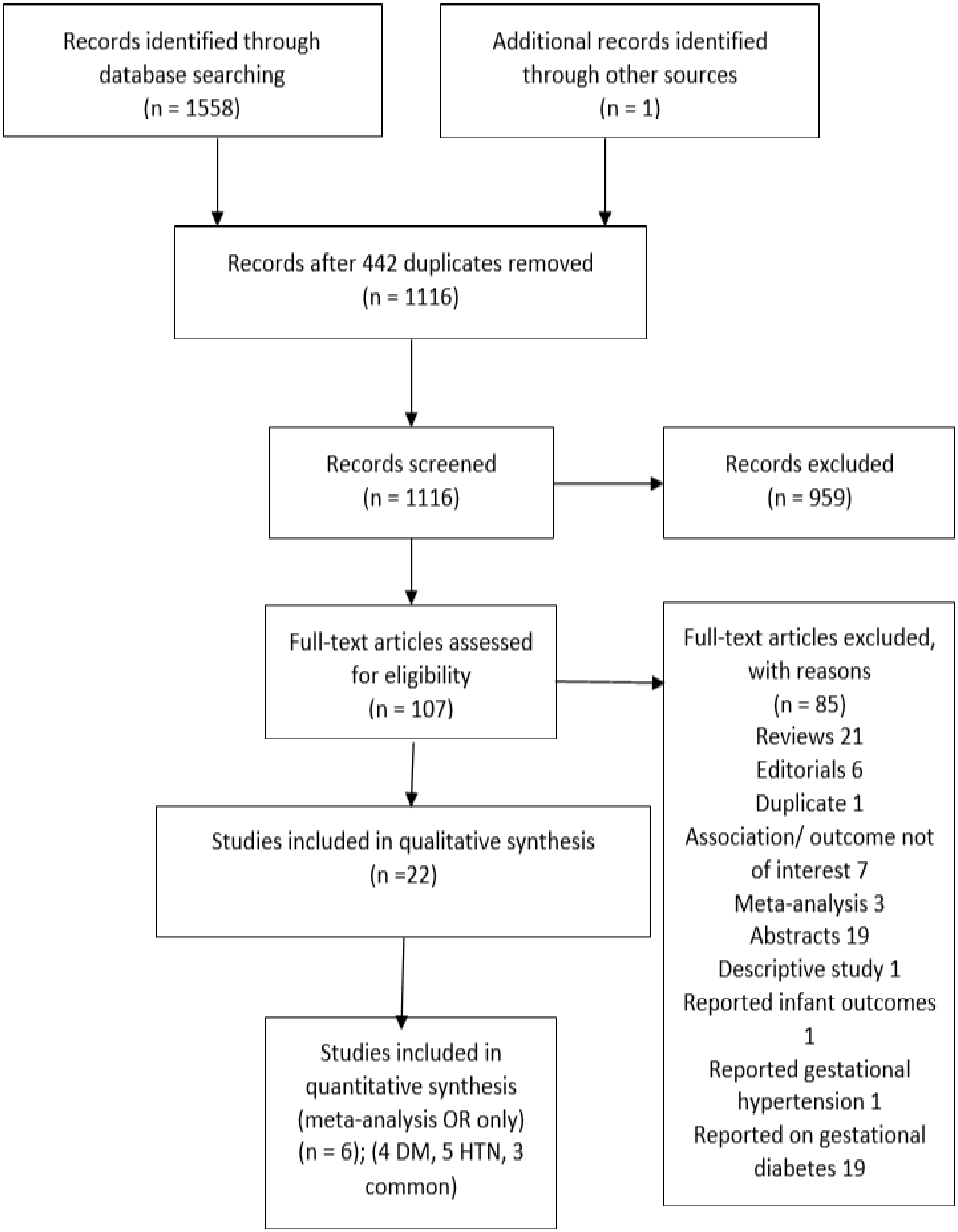

© 2019 Rameez RM et al. *JAMA Network Open.*

eTable 1: Assessment of quality of studies using the Newcastle-Ottawa scale

Name of study	Study type	Study outcome	Selection				Comparability		Outcome			Score
			Representative ness of the exposed cohort	Selection of the non-exposed cohort	Ascertainment of exposure	Demonstration that outcome of interest was not present at start of study	Study controls for lactation	Study controls for any additional factor	Assessment of outcome	Was follow-up long enough for outcomes to occur	Adequacy of follow up of cohorts	
**Liu, 2010**	Cross sectional	DM	*	*	*	*	*	*		*	*	8
**Schwarz, 2009**	Prospective	DM, HTN	*	*	*		*	*	*	*	*	7
**Zhang, 2015**	Cross sectional	DM, HTN	*	*	*		*	*	*			6
**Choi, 2017**	Cross sectional study	DM, HTN	*	*			*		*			4
**Chetwynd, 2017**	Cohort with nested case control	HTN	*	*	*	*	*	*		*		7
**Lupton, 2013**	Cohort	HTN	*	*	*	*	*	*		*		7

Quality of the included studies was appraised using the standardized New Castle Ottawa Scale(9). DM= diabetes, HTN = hypertension.

© 2019 Rameez RM et al. *JAMA Network Open.*

eFigure 2: Sub analysis with studies reporting RR, OR and unadjusted OR for breast feeding and diabetes:

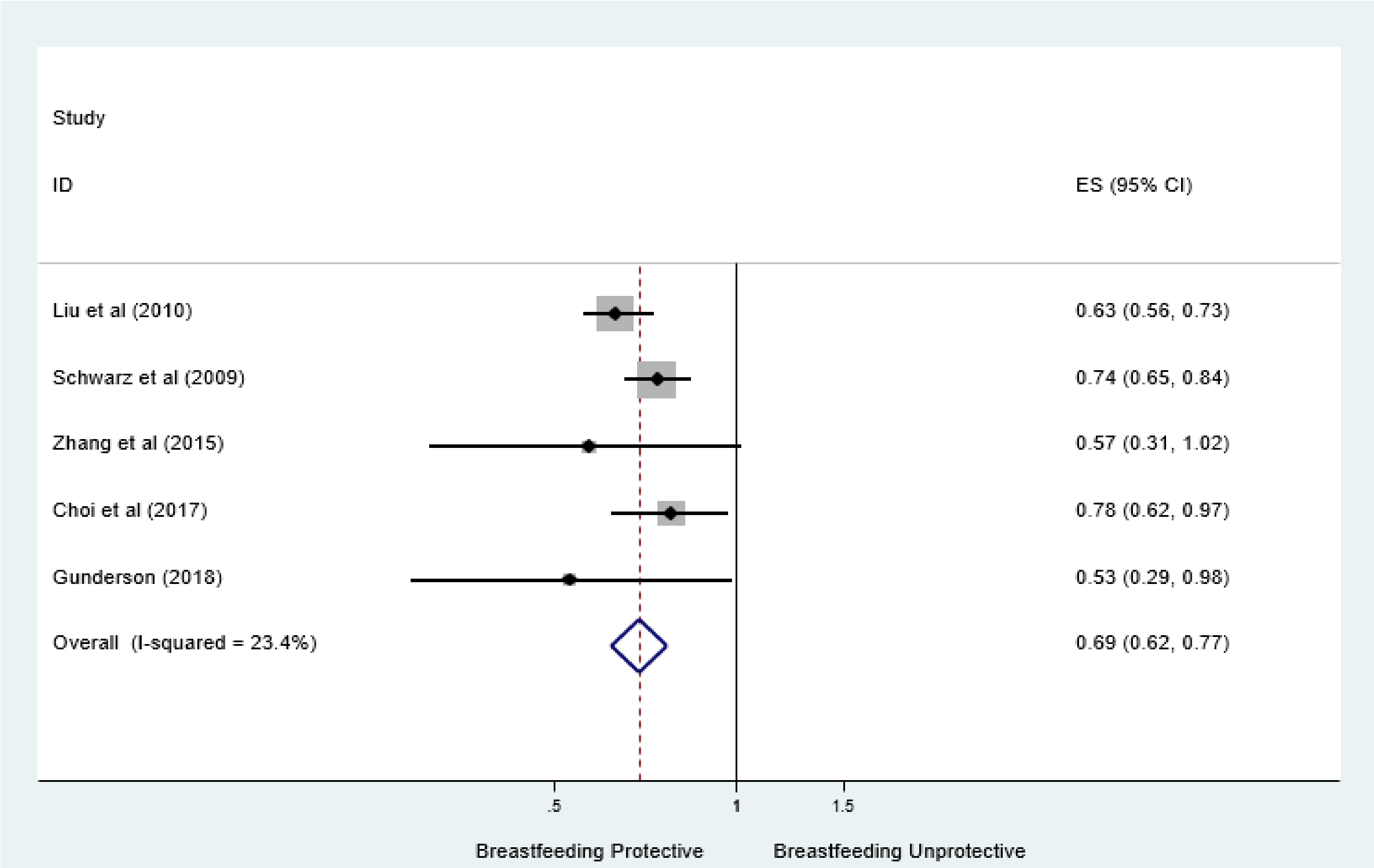

This sub-analysis pooled results of the studies that reported RR and OR and continued to show that breast feeding for more than 12 months was protective against diabetes.

© 2019 Rameez RM et al. *JAMA Network Open.*

eFigure 3: Sub analysis with studies reporting RR, OR, unadjusted OR and HR for breast feeding and diabetes:

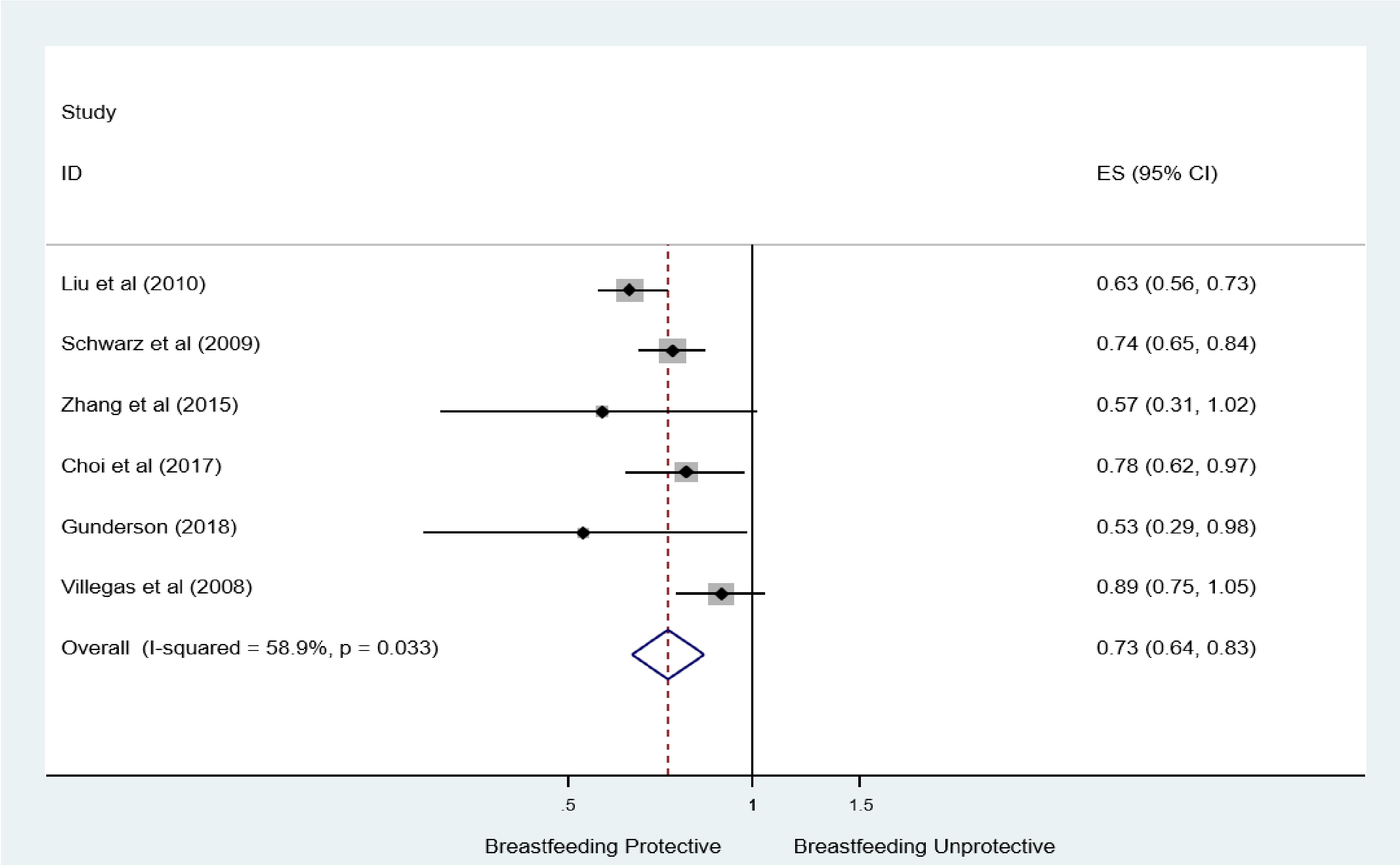

This sub-analysis pooled results of the studies that reported RR, OR, and HR, and continued to show that breast feeding for more than 12 months was protective against diabetes.

© 2019 Rameez RM et al. *JAMA Network Open.*

eFigure 4: Sub analysis with studies reporting RR, OR and unadjusted OR for breast feeding and hypertension:

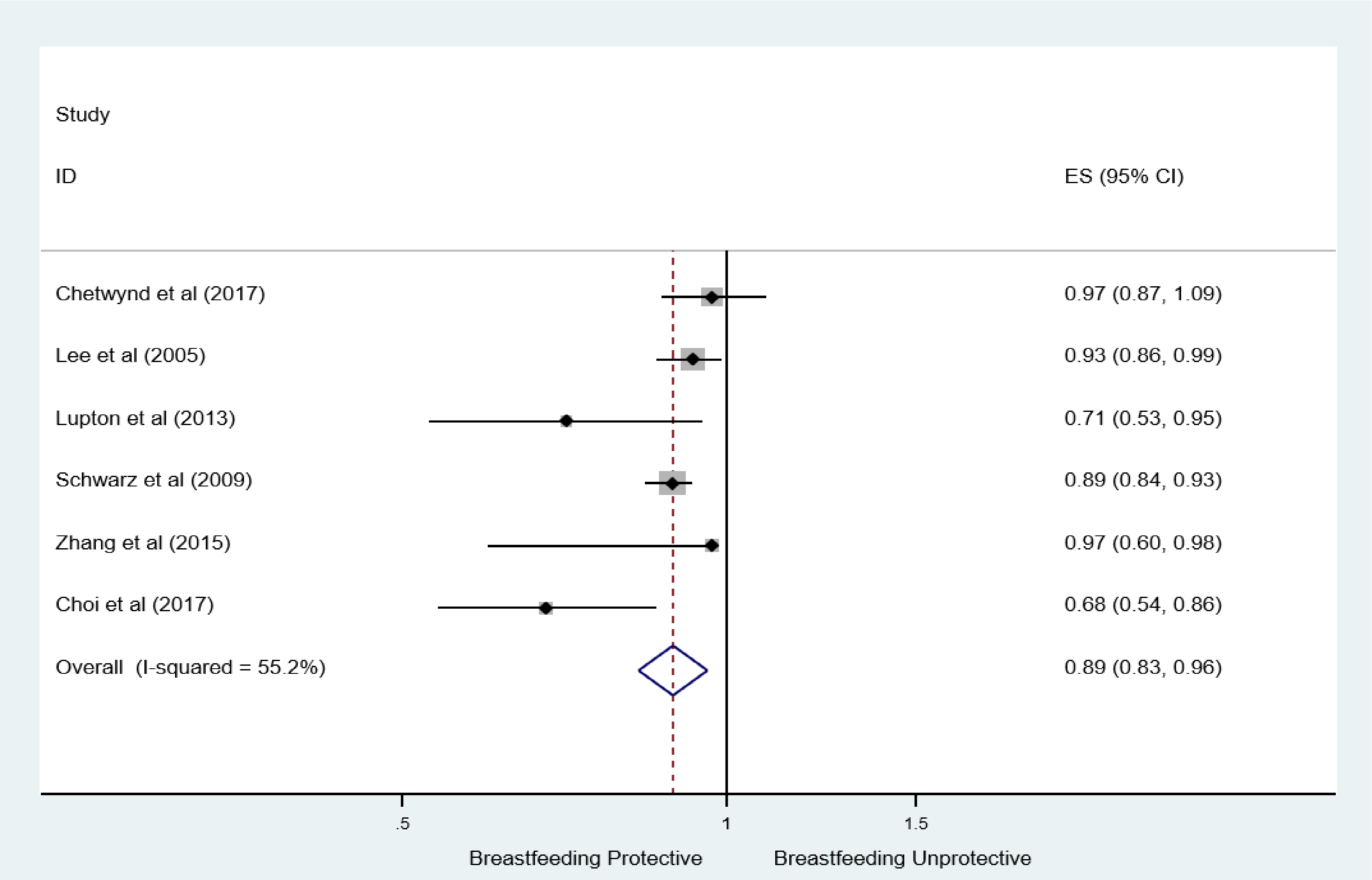

This sub-analysis pooled results of the studies that reported RR and OR, and continued to show that breast feeding for more than 12 months was protective against hypertension.

© 2019 Rameez RM et al. *JAMA Network Open.*

eAppendix: Detailed Search Strategy:

Misbah Systematic Review 7-3-18

Ovid Medline = 276
Ovid Embase with Conference Proceedings Abstracts = 884
CINAHL = 326
Cochrane CENTRAL = 72
Total = 1558
Duplicates removed = 442
Total to review = 1116

Database: Ovid MEDLINE(R) Epub Ahead of Print, In-Process & Other Non-Indexed Citations, Ovid MEDLINE(R) Daily, Ovid MEDLINE and Versions(R) <1946 to June 27, 2018> Search Strategy:

----

1 exp cardiovascular diseases/ or metabolic syndrome/ or diabetes mellitus, type 2/
2 risk/ or risk factors/ or risk assessment/ or incidence/ or prevalence/
3 1 and 2
4 ((cardiovascular or cardiac or hypertensi* or stroke or myocardi* or infarct* or cad or coronary or cvd or ischaemi* or ischemi* or cardiometaboli* or cardio-metaboli* or "metabolic syndrome" or "type 2 diabetes") adj5 (risk or risks or prevalence or incidence)).tw.
5 3 or 4
6 breast feeding/ or lactation/
7 ("breast feed*" or breastfeed* or "breast fed" or breastfed or lactation or lactating).tw.
8 6 or 7
9 5 and 8
10 women's health/ or maternal health/ or mothers/
11 (mother or mothers or maternal or woman or women or female or females).tw.
12 10 or 11
13 9 and 12
14 epidemiologic studies/ or exp case-control studies/ or exp cohort studies/ or controlled before-after studies/ or cross-sectional studies/ or multicenter studies as topic/ or double-blind method/ or random allocation/
15 exp clinical studies as topic/ or exp clinical study/
16 (clinical trial$1 or observational or longitudinal or prospective or retrospective).mp.
17 (controlled trial$1 or case-control* or cohort or follow-up).mp.
18 (cross-section* or multi-center or multicenter).mp.
19 (randomized or randomised or rct or rcts).mp.
20 (case series or registry or registries).mp.
21 ((compar* or evaluati* or validat*) adj3 (study or studies)).mp.
22 13 and (or/14-21)
23 limit 22 to english language
24 limit 23 to animals
25 limit 23 to (animals and humans)
26 24 not 25
27 23 not 26

Database: Embase, including Conference Proceedings Abstracts <1974 to 2018 July 02>
Search Strategy:

----

1 exp cardiovascular disease/
2 non insulin dependent diabetes mellitus/

© 2019 Rameez RM et al. *JAMA Network Open.*

3 1 or 2
4 risk/ or risk assessment/ or risk factor/
5 incidence/
6 prevalence/
7 4 or 5 or 6
8 3 and 7
9 cardiovascular risk/ or cardiometabolic risk/
10 ((cardiovascular or cardiac or hypertensi* or stroke or myocardi* or infarct* or cad or coronary or cvd or ischaemi* or ischemi* or cardiometaboli* or cardio-metaboli* or "metabolic syndrome" or "type 2 diabetes") adj5 (risk or risks or prevalence or incidence)).tw.
11 8 or 9 or 10
12 breast feeding/ or lactation/
13 ("breast feed*" or breastfeed* or "breast fed" or breastfed or lactation or lactating).tw.
14 12 or 13
15 women's health/ or maternal care/ or maternal welfare/ or mother/
16 (mother or mothers or maternal or woman or women or female or females).tw.
17 15 or 16
18 11 and 14 and 17
19 exp clinical study/ or comparative study/ or controlled study/ or observational study/
20 methodology/ or exp "clinical trial (topic)"/ or cohort analysis/ or control group/ or correlational study/ or cross-sectional study/ or double blind procedure/ or exp evidence based practice/ or triple blind procedure/
21 multicenter study/
22 (clinical trial$1 or observational or longitudinal or prospective or retrospective).mp.
23 (controlled trial$1 or case-control* or cohort or follow-up).mp.
24 (cross-section* or multi-center or multicenter).mp.
25 (randomized or randomised or rct or rcts).mp.
26 (case series or registry or registries).mp.
27 ((compar* or evaluati* or validat*) adj3 (study or studies)).mp.
28 18 and (or/19-27)
29 limit 28 to english language
30 limit 29 to animals
31 limit 29 to human
32 29 not 30
33 31 or 32

Cochrane CENTRAL, 1898 to date

#1 [mh "cardiovascular diseases"] or [mh "metabolic syndrome"] or [mh ^"diabetes mellitus, type 2"]
#2 [mh ^risk] or [mh "risk factors"] or [mh "risk assessment"] or [mh incidence] or [mh prevalence]
#3 #1 and #2
#4 (cardiovascular or cardiac or hypertensi* or stroke or myocardi* or infarct* or cad or coronary or cvd or ischaemi* or ischemi* or cardiometaboli* or cardio-metaboli* or "metabolic syndrome" or "type 2 diabetes") near/5 (risk or risks or prevalence or incidence)
#5 #3 or #4
#6 [mh "breast feeding"] or [mh lactation]
#7 "breast feed*" or breastfeed* or "breast fed" or breastfed or lactation or lactating
#8 #6 or #7
#9 #5 and #8
#10 [mh "women's health"] or [mh "maternal health"] or [mh mothers]
#11 mother or mothers or maternal or woman or women or female or females
#12 #10 or #11
#13 #9 and #12 in Trials

© 2019 Rameez RM et al. *JAMA Network Open.*

CINAHL Plus with Full Text, 1983 to date

S1 (MH "Cardiovascular Diseases+") OR (MH "Metabolic Syndrome X") OR (MH "Diabetes Mellitus, Type 2")
S2 (MH "Risk Assessment") OR (MH "Risk Factors") OR (MH "Incidence") OR (MH "Prevalence")
S3 S1 AND S2
S4 ((cardiovascular or cardiac or hypertensi* or stroke or myocardi* or infarct* or cad or coronary or cvd or ischaemi* or ischemi* or cardiometaboli* or cardio-metaboli* or "metabolic syndrome" or "type 2 diabetes") n5 (risk or risks or prevalence or incidence))
S5 (MH "Cardiovascular Risk Factors")
S6 S3 OR S4 OR S5
S7 (MH "Breast Feeding") OR (MH "Lactation")
S8 ("breast feed*" or breastfeed* or "breast fed" or breastfed or lactation or lactating)
S9 S7 OR S8
S10 S6 AND S9
S11 (MH "Women's Health") OR (MH "Maternal Welfare") OR (MH "Mothers+")
S12 (mother or mothers or maternal or woman or women or female or females)
S13 S11 OR S12
S14 S10 AND S13 Limiters - English Language

© 2019 Rameez RM et al. *JAMA Network Open.*

eTable 2: Qualitative analysis of 18 studies

Name of first author, year published	Type of study	Total participants	Follow up (years)	Age group (years)	Region/ country	Race/ ethnicity	Outcome (HTN, DM)	Time point for outcomes (years)	Outcome assessment	Adjusted covariates	Study inclusion criteria	Study exclusion criteria	Reason for exclusion
**Stuebe, 2005**	Longitudinal cohort, prospective cohort study; retrospective analysis of NHS2			25-42	USA	Not specified	DM	Not available	Direct measurement, classic symptoms, currently using medication	Age, parity, body mass index at age 18 years, diet, physical activity, family history of diabetes, and smoking status	Parous women without prevalent HTN, diabetes, cardiovascular disease, hyperlipidemia or cancer	Not available	Did not meet full inclusion criteria of breast feeding >12 months
**NHS1**		83,585	16; (1 239 709 person years of follow-up)										
**NHS2**		73,418	12; (778876 person years of follow-up)										

© 2019 Rameez RM et al. *JAMA Network Open.*

**Lee, 2005**	Population cohort	177,749	6	20-59	Seoul, Korea	Asian	HTN	Not available	Direct measurement or use of medication	Adjusted for age, obesity, smoking, alcohol drinking, exercise, number of children, and age at first pregnancy	Age >=20, premenopausal	Not available	Report outcome as RR, included in sub-analysis
**Villegas, 2008**	Prospective cohort	62,905	4.6	40-70	China	Asian	DM	Variable, 2-13 years since last pregnancy	Measured or if self-reported, confirmed by review of medical records, blood tests or use of medication	Age, BMI, waist hip ratio, number of live births	Age 40-70, no prior history of DM, cancer or cardiovascular disease	Not available	Reported outcomes as RR, included in sub-analysis
**Ram, 2008**	Cross sectional cohort analysis	2,516	2	42-52	USA	Not available	DM, HTN	Not available	Direct measurement	Adjusted for age, smoking history, parity, ethnicity, study site, socioeconomic status, physical activity, daily caloric intake, high school BMI	Age 42-52, intact uterus, at least one ovary, at least one menstrual period within the last 3 months, no reproductive hormones within the last 3 months, at least one live birth	Not mentioned	Breast feeding categories of ever vs never, does not meet inclusion criteria of at least 12 months breast feeding
**Shwarz, 2010**	Prospective cohort study	2,233	5	40-78	USA	40% White, 18% Asia, 20% African	DM	Not available	Direct measurement, self-report or use of medication	Age, parity, race, education, income, physical activity, tobacco and alcohol use	Never pregnant, one or more live births with complete lactation data	Women with missing information for parity, lactation and those who only had still births	Breast feeding category of >1 month, did not meet inclusion criteria

© 2019 Rameez RM et al. *JAMA Network Open.*

**Steube, 2010**	Prospective cohort study	566	3	35-40	USA	Asian, black, Hispanic, white and other	DM	3 years post-partum	Direct measurement, blood tests	Adjusted for pre-pregnancy BMI, smoking status, intention to lose weight, dietary intake, physical activity, hormone replacement therapy	Fluent in English, <22 weeks' gestation at study entry, singleton pregnancy	Not delivered another child since delivery of index child at 3 years, no prior diabetes, follow up for at least 3 years, missing data	Results reported as mean HBA1c, could not be analyzed
**Stuebe, 2011**	Observational cohort	55,636	14	25-42	USA	White, Black, Hispanic, Asian and other	HTN	Not available	Self-reported, verified by medical records	Age, weight, self-reported history of preeclampsia, gestational hypertension, gestational diabetes, birth of an infant at <37 weeks' gestation, birth of an infant weighing <2,500 g, miscarriage or stillbirth at >12 weeks' gestation, smoking status, vigorous physical activity, alcohol consumption, Dietary Approaches to Stop Hypertension (DASH) diet score quintile, family history of hypertension, current oral contraceptive use, current nonnarcotic analgesic use, and self-reported race.	Not available	Nulliparous and those missing data on breast feeding, those reporting HTN, DM, HLD, cardiovascular disease and cancer prior to start of data collection	No value reported for breast feeding duration >12 months, which was used as a reference

© 2019 Rameez RM et al. *JAMA Network Open.*

**Jäger 2014**	Case cohort	1,059	11	35-64	Germany	Not available	DM	Not available	Self-reported, confirmed by treating physician	Age at baseline, marital status, education, occupation, smoking, sport, cycling, alcohol intake, coffee consumption, intake of red meat, intake of whole-grain bread, BMI at base line examination, waist circumference at base line examination	Not available	Missing data on breast-feeding behavior, oral contraceptive use, implausible energy intake; prevalent diabetes including gestational diabetes, women with missing data on BMI at the age of 25 years and missing biomarker measurements	Breast feeding >6 months which does not meet inclusion criteria of breast feeding for at least 12 months

© 2019 Rameez RM et al. *JAMA Network Open.*

**Gunderson, 2015**	Prospective	846	20	18–30	USA	52% black, 48% white	DM, atherosclerosis, carotid intima media thickness	Variable	Direct measurement	Pre-pregnancy measures (BMI, HDL-C, blood pressure, homeostatic model assessment of insulin resistance), parity, education, age, number of postbaseline births, race, smoking, and time since last birth	Without heart disease or overt diabetes before pregnancy, delivered at least once, reported lactation duration	Women who did not attend 20 year follow up, who did not have carotid artery thickness measurement; history of heart disease, recent or current pregnancy, hysterectomy or removal of both ovaries at baseline, diabetes at baseline, no post baseline births, missing lactation information	Main study outcome is development of atherosclerosis
**Bajaj, 2016**	Prospective cohort	330	3	Mean age 35 +/-4	Canada	NA	DM	Up to 3 years post-partum	Direct measurement using oral glucose tolerance tests	Age, ethnicity, family history of diabetes, prepregnancy BMI, and months postpartum	Postpartum women with available information on breast feeding practices	Not available	Report outcomes as RR, included in sub-analysis
**Martens, 2016**	Retrospective cohort	334,553	24	mean age 25-27	Canada	86% non-First nations, 56% First Nation	DM, pre-DM	Variable	Hospitalization or outpatient visits with diagnosis of DM	Not available	All women with live births in a hospital in Manitoba between 1987 and 2011, without prepregnancy diabetes	Women with a diagnosis of gestational diabetes or incident diabetes within the first 20 weeks of gestation	Outcome was pre-DM and DM; also report raw data which cannot be adjusted for covariates

© 2019 Rameez RM et al. *JAMA Network Open.*

**Moradi, 2016**	Cross sectional	978	Not available	40-70	Iran	Persian	HTN, DM, MetS	Not available	Direct measurement	Age, age at first pregnancy, number of pregnancies	Age 40-70, at least one live birth history, referred to health centers of Amol in Iran	Not available	Primary outcome was metabolic syndrome
**Kim, 2016**	Cross sectional	1,053	Not available	30-49	Korea	Asian	HTN, MetS	Not available	Direct measurement	Not available	Females of child bearing potential, at least one prior birth	History of stroke, myocardial infarction, angina, renal failure, cancer	Breast feeding reported as absent or present without specified duration
**Park, 2017**	Cross sectional	3,119	1	>=50	South Korea	Asian	HTN	Not available	Direct measurement	Adjusted for age, alcohol consumption, regular physical activity, education level, income, obesity, DM, number of pregnancies	Not mentioned	Nulliparity, current smokers, menopausal	Do not give data for breast feeding 12 months
**Gunderson, 2018**	Prospective cohort study	1,238	30	18-30	USA	White and Black	DM	Not available	Direct measurement, self-report or use of medication	Race, prepregnancy, BMI, waist circumference, fasting blood glucose, age, time-dependent parity and gestational DM status, family history of diabetes	Age 18-30, without DM at baseline, 1 or more live births, reported lactation duration	Those with diabetes, hysterectomy, oophorectomy and with pregnancy or lactation at baseline	Reports outcomes as HR, included in sub-analysis

© 2019 Rameez RM et al. *JAMA Network Open.*

**Kirkegaard, 2018**	Prospective cohort	63,260	6	Average age 29.8-45.9	Denmark	Not available	HTN	Not available	Review of medical records	Age, prepregnancy BMI, alcohol intake before the index pregnancy, socio-occupational status, dietary intake, physical activity, smoking, preterm birth, preeclampsia and diabetes during the index pregnancy and parity at 18 months postpartum.	Women with at least one live born singleton	Prior history of HTN, DM, ischemic heart disease, stroke or other cardiovascular disease diagnosis before start of follow up, those missing breast-feeding information, those who were pregnant at 7 years or within 12 months post-partum at the time of follow up, missing prepregnancy BMI	Breast feeding category of >10 months which does not meet inclusion criteria of at least 12 months of breast feeding

BMI = body mass index, DM = diabetes, HTN = hypertension, MetS = metabolic syndrome.

© 2019 Rameez RM et al. *JAMA Network Open.*
